# Construction and Psychometric Evaluation of the Critical Thinking Disposition Assessment Questionnaire Among Medical Undergraduate Students in West Bengal, India

**DOI:** 10.7759/cureus.70266

**Published:** 2024-09-26

**Authors:** Diptakanti Mukhopadhyay, Sonali G Choudhari

**Affiliations:** 1 Department of Community Medicine, College of Medicine & Sagore Dutta Hospital, Kolkata, IND; 2 Department of Community Medicine, Jawaharlal Nehru Medical College, School of Epidemiology and Public Health, Datta Meghe Institute of Higher Education & Research, Wardha, IND

**Keywords:** critical thinking disposition, india, psychometrics, questionnaire, undergraduate medical students

## Abstract

Introduction

Assessing the critical thinking disposition of students with a valid tool in an Indian setting was a prerequisite to introducing critical thinking under the undergraduate medical curriculum, as it was shaped by society, culture, and language. The study planned to design and assess the psychometrics of the Critical Thinking Disposition Assessment Questionnaire (CTDAQ) in the Indian setting.

Methods

The study was conducted among undergraduate students at a medical college in West Bengal, India. A 30-item scale was initially designed through the review of relevant literature and discussion with experts. Through two rounds of the Delphi technique, a 20-item CTDAQ was formulated, and the content validity index (CVI) was calculated. The test-retest agreement was calculated after administering the tool twice among 25 students with a gap of three weeks using the weighted kappa coefficient. Then, principal component analysis was conducted to extract component structure with rotation. The internal consistency was measured using Cronbach’s alpha.

Results

Item-wise and scale CVIs were more than 0.80 and 0.90, respectively. All items had a weighted kappa coefficient of ≥0.75. A three-component model was extracted from 20 items, with a minimum factor loading of 0.30. It explained 50.3% of the total variance. Cronbach's alpha of the scale, the *conversance and systematicity* component with seven items, the *analyticity and flexibility* component with nine items, and the *maturity* component with four items were 0.783, 0.789, 0.825, and 0.797, respectively.

Conclusions

The 20-item CTDAQ is psychometrically a valid, stable, and internally consistent scale to assess critical thinking disposition in the Indian context.

## Introduction

In a consensus statement, critical thinking in the context of health professions education was defined as "the application of higher cognitive skills (e.g., conceptualization, analysis, evaluation) to information (gathered from medical history, records, physical examination, or diagnostic investigation) in a way that leads to action that is precise, consistent, logical, and appropriate” [1]. It was adapted from the definition of critical thinking by Scriven and Paul [2]. In clinical practice, widespread uncertainty prevails in defining and diagnosing the disease, assessing potential outcomes of disease management procedures, and making a management decision [3,4]. Critical thinking helps assess the credibility of information, adapt to problems, simplify communication and coordination, and make effective decisions [3-6].

Biomedical knowledge and cognitive processing skills have interdependence [5]. Collectively, both contributed to clinical reasoning skills, which are necessary for making diagnostic and management decisions [7,8]. Critical thinking, the process of scrutinizing and appraising thinking to make decisions, helps clinical reasoning understand the effect of personal views, beliefs, and biases on clinical decision-making [2]. Those skills helped in developing conceptual understanding and metacognition and, as a result, also helped in problem-solving during clinical practice [7].

Although the role of critical thinking for medical practitioners was emphasized, the framework of core competencies in undergraduate medical curricula did not include it explicitly [3,9]. It is imperative to have a tool to objectively measure the critical thinking of medical students for incorporating critical thinking in teaching and learning within the ambit of the undergraduate medical curriculum. As per published literature, there are two dimensions of critical thinking: disposition and skills [9]. Disposition reflects how individuals approach a task [10]. To foster the ability of critical thinking, students should be predisposed to embrace complexities and be open to uncertainty [10]. There are a few tools in English to assess critical thinking disposition, such as Watson and Glaser Critical Thinking Appraisal (WGCTA), California Critical Thinking Disposition Instrument (CCTDI), and Critical Thinking Disposition Scale, which were primarily used in Western cultures [11,12]. Similarly, a number of questionnaires were developed in Asian countries for this purpose and validated among healthcare students or professionals [13,14]. However, due to trans-cultural variation, it was imperative to establish the psychometric properties of the tool for its use in the Indian socio-cultural setting. As per available published literature, no such tool was found, even after a diligent search.

In this context, this study was planned at a medical college in Kolkata, West Bengal, to construct and evaluate the psychometric properties of the Critical Thinking Disposition Assessment Questionnaire (CTDAQ) in English.

## Materials and methods

A cross-sectional study was conducted during November- December 2023 among the undergraduate medical students of a Medical College in West Bengal, India [15].

The CTDAQ was initially designed by reviewing relevant published literature, the available scales, and informal discussions with subject material experts [10-14]. The taxonomies of thinking dispositions proposed by different authors were reviewed [11]. In the next step, a consensus was built between the authors to select the dispositions that were considered essential for critical thinking. During this process, five subject material experts from the disciplines of psychiatry, psychology, and medical education were consulted to get further clarification. The dispositions to be alert about the context, to collect information from all possible sources, to arrange the data orderly, to analyze the collected data, to be flexible based on the context, intention to make decisions based on the data and the context, and the intention to review own assumptions and decisions were considered during framing of questions. Those were distributed across the critical thinking dispositions proposed by different researchers [10-14]. Several dispositions such as self-confidence, concerns for others’ welfare, and abilities were not included during the selection of items of the present scale. The initial list of questions was reviewed by the authors, and in the light of discussions with the five subject material experts, 30 items were retained after refinement and removal of duplicate items.

Considering its potential use in medical education, the questionnaire was developed in English. Simple language was used for better understanding. As the language of instructions and assessment in medical education is primarily English in India, it was assumed that the questionnaire will be comprehensible to all students and experts. A five-point Likert scale was employed to rate each item, ranging from 1 for strong disagreement to 5 for strong agreement. The reverse scoring pattern was followed for the negative items. The score of all constituent items would be added to obtain total and component-wise scores. The higher the score, the higher the critical thinking disposition would be considered.

The first version, which had 30 items, was shared with nine subject material experts from psychiatry, psychology, and medical education, with the request to rate the items as essential, or desirable, or non-essential. They were also requested to suggest modifications in the framing and wording of the items and additional items they considered crucial. The items that were considered as either essential or desirable by eight out of nine experts were retained. The second version of the questionnaire with 20 items was designed using two rounds of the Delphi technique.

The second version was pretested among 10 undergraduate medical students to check the comprehensibility, acceptability, and flow of questions. Based on their feedback, the third version of the questionnaire was developed. Then, it was sent to nine subject material experts to measure content validity. Content validity indices (CVIs) for individual items and the scale were calculated [16,17]. Based on CVIs, all 20 items were retained in the fourth version [16,17].

The fourth version was then administered twice among 25 first-year students with a gap of three weeks. Weighted kappa statistics were used to assess the test-retest agreement of each item. Then, it was sent to 220 medical students. Out of them, 201 students responded. The exploratory principal component analysis was conducted with data from 201 students to establish construct validity with varimax rotation. The internal consistency of the entire scale and individual components was calculated using Cronbach's alpha [15,18,19].

The extraction of factor structure through principal component analysis followed Horn’s parallel analysis as it was considered more accurate in deciding the number of components to be retained [18,20]. The minimum factor loading was 0.3, as it was of practical significance and allowed the retention of a sufficient number of items [19].

The data were collected through a Google Form shared on the WhatsApp of the individual students. A period of 72 hours was allowed to fill up the forms. The informed consent form was incorporated in the first section of the Google Form. Only those students who consented to participate could proceed to the next section. The following section intends to collect socio-demographic and individual information anonymously. The last section consisted of the 20-item CTDAQ. Age was recorded in completed years. Subjective economic status was assessed on a scale of 5: (1) “very well off,” (2) “living comfortably,” (3) “just getting by,” (4) “almost poor,” and (5) “poor” [21].

The data gathered through Google Forms were downloaded into an MS Excel spreadsheet. At first, the consistency of the data was checked. The weighted kappa was calculated using R Statistical Software version 4.4.0 (package: irr, R Foundation for Statistical Computing, Vienna, Austria). All other tests were conducted using the Jamovi 2.4.11 software. The selection of components was based on the parallel analysis method and scree plot [19]. The model fit was assessed using Kaiser-Meyer-Olkin (KMO) statistics and Bartlett's test for sphericity [18,19].

The concerned Institutional Ethics Committee approved the study (Ref No. CMSDH/IEC/19/03-2023; date: 27.03.2023). After communicating about the study and their ethical rights, the participants were requested to provide written informed consent through Google Forms.

## Results

Out of the 220 students who were approached, 201 (91.4%) participated in the study. Among the participants, 83 (41.3%) were females and the rest were males. The mean (± SD) age of the study participants was 21.5 (± 2.5) years. On inquiry about subjective economic status, 127 (63.2%) reported being very well off or living comfortably, whereas 17 (8.5%) were poor or almost poor.

Content validity and test-retest agreement

Based on structured responses of nine subject material experts, the item-wise content validity index (I-CVI) for 20 items was ≥0.89, and the average scale content validity index (S-CVI/Ave) was 0.98 for both clarity and relevance separately. The weighted kappa value for all items except items 4, 5, 7, and 17 was ≥0.80, whereas the weighted kappa coefficients for those four items were ≥0.75.

Exploratory principal component analysis

As shown in Table 1, three principal components were identified based on the parallel analysis method and scree plot. Items with factor loadings in more than one component were included in the component where it showed the highest factor loading. The first component with seven items (1, 2, 3, 4, 5, 11, and 15) was named conversance and systematicity. The second component with nine items (6, 7, 8, 10, 13, 16, 17, 18, and 20) was named analyticity and flexibility. The third component with four items (9, 12, 14, and 19) was named maturity. The three components accounted for 50.3% of the explained variances. Bartlett’s test of sphericity (χ2= 1565; df=190; p<0.001) was significant. The overall KMO measurement of sampling adequacy was 0.805. Both the statistics established the applicability of principal component analysis.

**Table 1:** Item-wise factor loading after principal component analysis with varimax rotation *Cells without any numerical values signified factor loading <0.3

Item No.	Item Statement	Conversance and Systematicity*	Analyticity and Flexibility*	Maturity*
1	I always make every effort to collect all the relevant information before making an important decision	0.482	0.367	-
2	I try to dig out all features of any viewpoint that is new and novel to me	0.450	0.350	-
3	I organize all the collected information systematically to reach a rational decision	0.654	-	-
4	In times of emergencies, I strive to seek all available solutions before deciding the best	0.687	-	-
5	When encountered with an unknown event, I try to assess information to understand the event	0.599	0.301	-
11	During the discussion, I usually follow well-defined logic	0.685	-	-
15	I usually do not change from the pre-decided plan of action	0.595	-	-
6	Before making a judgment, I am used to analyze all the available information and the prevailing situation	0.387	0.584	-
7	I always assess the value of every piece of information before drawing reasonable conclusions and making a decision	0.407	0.585	- 0.318
8	I try to comprehend every detail of the problem that may have a relation to the given problem	0.368	0.669	-
10	I always examine the pros and cons of each available option to solve the problem	0.411	0.566	-
13	I usually listen to and try to understand different viewpoints before expressing my opinion.	-	0.572	-
16	I willingly accept criticism of my opinion	- 0.313	0.648	-
17	I never hesitate to question my prejudices, assumptions, beliefs, or past decisions.	-	0.589	-
18	I consider my mistakes as an opportunity to learn	-	0.512	-
20	I use logic and reasoning while dealing with complicated problems	-	0.641	-
9	I intend to solve the problem without considering all the details of the problem	-	-	0.760
12	I tend to be influenced by the opinions of other participants	-	-	0.766
14	I find it difficult to alter my viewpoints even though there is evidence to challenge it	-	-	0.768
19	The pre-fixed, subjective idea about an event or the concerned person influences my decision	-	-	0.816

Internal consistency

As shown in Table 2, Cronbach's alpha for the entire scale was 0.783, whereas the values for three subscales, namely conversance and systematicity with seven items, analyticity and flexibility with nine items, and maturity with four items, were 0.789, 0.825, and 0.797, respectively. Cronbach's alpha in any of the subscales was not increased with the dropping of any item.

**Table 2:** Cronbach’s alpha of each component and the scale

Scale/Subscale	Mean	SD	Cronbach’s alpha
CTDAQ	3.78	0.35	0.783
Conversance and systematicity	3.99	0.47	0.789
Skepticism and analyticity	3.97	0.48	0.825
Maturity	2.96	0.77	0.797

## Discussion

Critical thinking disposition is defined as attributes that influence an individual's beliefs and actions [10,22]. CTDAQ was a 20-item scale developed and validated in an Indian socio-cultural setting. A robust assessment method was a prerequisite for incorporating critical thinking explicitly within the framework of the undergraduate medical curriculum. The present study was a step forward toward that goal. Constructing and evaluating psychometric properties of a new tool was necessary as the existing tools might not show similar psychometric properties during cross-cultural validation [23-25]. The language, culture, and perception of people shaped the disposition aspect of critical thinking [25].

Polit and Beck defined content validity as the “degree to which an instrument has an appropriate sample of items for the construct being measured” [16]. The S-CVI/Ave and the I-CVI of this questionnaire surpassed the proposed minimum values of 0.90 and 0.78, respectively [16,17]. The translated versions of CCTDI showed lower CVI compared to the English version [24,25]. Most of the published studies did not report CVI, whereas one study from Vietnam reported CVI in an acceptable range [11,14].

Internal consistency “reflects the coherence of the items in the component of scale” [26]. Test-retest reliability demonstrates “the extent to which almost similar scores are obtained when the scale is administered on different occasions” separated by a “wash-out period” [26]. Both contributed to the reliability of any scale, which is now considered evidence of validity [26]. According to earlier literature, weighted kappa was considered a dependable measure of test-retest agreement, where an ordinal scale was used [27]. The kappa values of all items of CTDAQ reflected their excellent stability [27]. Although several alternative measurements were available, Cronbach's alpha was commonly used in all new tools as it provided a robust estimate of internal consistency [28]. Cronbach’s alpha values of the three subscales of CTDAQ were within the range of 0.70 to 0.95, which indicated acceptable internal consistency [28,29]. Several other studies on developing a new critical thinking disposition scale across the globe reported acceptable alpha values [11,13,14,23].

Bartlett's test and the KMO statistics were comparable with similar studies, and the KMO statistics in this study were higher than the cut-off of 0.60 [13,24]. The three-component model of CTDAQ with 20 items was stable, and appropriate factor loadings were observed. Similar findings were observed from earlier research [13]. The first component, i.e., *conversance and systematicity*, reflected the inquisitiveness to collect all possible information about an event or the context and the ability to organize them following well-thought-out logic [13,23]. The second component, i.e., *analyticity and flexibility*, conveyed the ability to analyze collected information and to remain context-specific [13,23]. The third component, i.e., *maturity*, consisted of items reflecting the ability to make appropriate decisions based on available information in the given context. [13,23] These three components captured a number of perspectives that were related to the different concepts of critical thinking dispositions, such as “inquisitiveness,” “open-mindedness,” “systematicity,” “analyticity,” “truth-seeking,” “self-confidence,” and “maturity” [2,3,23].

The CTDAQ may be useful for educators and researchers to measure the disposition of undergraduate medical students towards critical thinking in Indian settings. At an individual and collective level, this questionnaire may be used to screen students to find areas of critical thinking disposition that need nurturing and feedback [11]. The CTDAQ may provide a framework for further research and alternative measures of critical thinking disposition. The effect of dispositions to critical thinking on different domains of learning and clinical practice needed further exploration [11]. However, this tool measures disposition to critical thinking rather than actual skill set. Moreover, other theoretical and/or empirical constructs proposed by earlier researchers to measure critical thinking disposition are not ruled out by this model [1,2,10,14,30].

However, the present study might suffer from the inadequacies of other self-reported questionnaire-based studies. The responses of the participants might be influenced by social desirability and individual emotion. Anonymity and confidentiality might act as a deterrent to those. The study lacked generalizability as it was conducted among the students of a medical college. Refinement of the scale with a larger representative sample from different parts of India would further add to the reliability and validity of the scale. The present study dealt with dispositions to critical thinking. However, for comprehensive assessment of critical thinking, the skill dimension may be brought under review.

## Conclusions

The CVI, as appraised by nine subject material experts, was fair as per existing standards. The test-rest agreement, as evident from weighted kappa statistics, confirmed the excellent stability of the questionnaire. The component structure of this 20-item questionnaire elicited three distinct components. The internal consistencies for the entire scale and the components were satisfactory. The homogeneity of the items under each component and the higher correlation between components and the contributing items were emphasized by higher values of Cronbach’s alpha. Based on this information, it may be inferred that CTDAQ can be a useful tool for measuring the dispositions of medical students to critical thinking in the Indian setting. Further research using CTDAQ in different settings would help establish the psychometric properties of the scale.

## References

[1] Huang GC, Newman LR, Schwartzstein RM (2014). Critical thinking in health professions education: summary and consensus statements of the Millennium Conference 2011. Teach Learn Med.

[2] Scriven Scriven, M M, Paul Paul, R R (2024). Defining Critical Thinking: Foundation for Critical Thinking. https://www.criticalthinking.org/pages/defining-critical-thinking/766.

[3] Zayapragassarazan Z, Menon V, Kar S, Batmanabane G (2016). Understanding critical thinking to create better doctors. J Adv Med Educ Res.

[4] Sharples JM, Oxman AD, Mahtani KR (2017). Critical thinking in healthcare and education. BMJ.

[5] Stanton NA, Wong W, Gore J, Sevdalis N, Strub M (2011). Critical thinking. Theor Issues Ergon Sci.

[6] Benner P, Hughes RG, Sutphen M (2008). Clinical reasoning, decision making, and action: thinking critically and clinically. Patient Safety and Quality: An Evidence-Based Handbook for Nurses.

[7] Scott IA, Hubbard RE, Crock C, Campbell T, Perera M (2021). Developing critical thinking skills for delivering optimal care. Intern Med J.

[8] Victor-Chmil J (2013). Critical thinking versus clinical reasoning versus clinical judgment: differential diagnosis. Nurse Educ.

[9] Krupat E, Sprague JM, Wolpaw D, Haidet P, Hatem D, O'Brien B (2011). Thinking critically about critical thinking: ability, disposition or both?. Med Educ.

[10] Ku KYL, Ho IT (2010). Dispositional factors predicting Chinese students’ critical thinking performance. Personal Individ Differ.

[11] Sosu EM (2013). The development and psychometric validation of a critical thinking disposition scale. Think Ski Creat.

[12] Sommers CL (2018). Measurement of critical thinking, clinical reasoning, and clinical judgment in culturally diverse nursing students - a literature review. Nurse Educ Pract.

[13] Cui L, Zhu Y, Qu J, Tie L, Wang Z, Qu B (2021). Psychometric properties of the critical thinking disposition assessment test amongst medical students in China: a cross-sectional study. BMC Med Educ.

[14] Shin H, Park CG, Kim H (2015). Validation of Yoon's Critical Thinking Disposition Instrument. Asian Nurs Res (Korean Soc Nurs Sci).

[15] Mukhopadhyay DK, Choudhari SG (2024). Critical thinking and clinical reasoning in undergraduate medical course: a mixed-methods study in a medical college in Kolkata, West Bengal, India. F1000Res.

[16] Polit DF, Beck CT (2006). The content validity index: are you sure you know what's being reported? Critique and recommendations. Res Nurs Health.

[17] Yusoff MSB (2019). ABC of content validation and content validity index calculation. Educ Med J.

[18] Henson RK, Roberts JK (2006). Use of exploratory factor analysis in published research: common errors and some comment on improved practice. Educ Psychol Meas.

[19] Kahn JH (2006). Factor analysis in counseling psychology research, training, and practice: principles, advances, and applications. Couns Psychol.

[20] Horn JL (1965). A rationale and test for the number of factors in factor analysis. Psychometrika.

[21] Gore S, Aseltine RH Jr, Colton ME (1992). Social structure, life stress and depressive symptoms in a high school-aged population. J Health Soc Behav.

[22] Perkins DN, Jay E, Tishman S (1993). Beyond abilities: a dispositional theory of thinking. Merrill-Palmer Q.

[23] Wang X, Sun X, Huang T, He R, Hao W, Zhang L (2019). Development and validation of the critical thinking disposition inventory for Chinese medical college students (CTDI-M). BMC Med Educ.

[24] Luiz FS, Leite ICG, Carvalho PHB de (2021). Validity evidence of the critical thinking disposition scale, Brazilian version. Acta Paul Enferm.

[25] Iskifoglu G (2014). Cross-cultural equivalency of the California critical thinking disposition inventory. Educ Sci Theory Pract.

[26] McCrae RR, Kurtz JE, Yamagata S, Terracciano A (2011). Internal consistency, retest reliability, and their implications for personality scale validity. Pers Soc Psychol Rev.

[27] Fleiss Fleiss, JL JL, Levin Levin, B B, Paik Paik, MC MC (2003). Statistical Methods for Rates and Proportions.

[28] Tavakol M, Dennick R (2011). Making sense of Cronbach's alpha. Int J Med Educ.

[29] Bland JM, Altman DG (1997). Cronbach's alpha. BMJ.

[30] Ennis Ennis, RH RH (1991). Critical thinking: a streamlined conception. Teach Philos.

